# Fatigue Strength Assessment of Friction Welds under Consideration of Residual Stress

**DOI:** 10.3390/ma17133130

**Published:** 2024-06-26

**Authors:** Lorenz Uhlenberg, Jörg Baumgartner, Christoph Rößler, David Schmicker, Markus Köhler, Frank Trommer, Klaus Dilger

**Affiliations:** 1 Institute of Joining and Welding, Technische Universität Braunschweig, Langer Kamp 8, 38106 Braunschweig, Germany; markus.koehler@tu-braunschweig.de (M.K.); k.dilger@tu-braunschweig.de (K.D.); 2 Fraunhofer Institute for Structural Durability and System Reliability LBF, Bartningstr. 47, 64289 Darmstadt, Germany; joerg.baumgartner@lbf.fraunhofer.de; 3 Sampro GmbH, Dillweg 17b, 39110 Magdeburg, Germany; christoph.roessler@sampro-software.com (C.R.); david.schmicker@ifa-group.com (D.S.); 4 Institute of Mechanical Engineering, Hochschule Magdeburg-Stendal, Breitscheidstraße 2, 39114 Magdeburg, Germany; frank.trommer@h2.de

**Keywords:** rotary friction welding, residual stress, fatigue, structural steel, numerical modelling, fatigue strength assessment

## Abstract

A reliable local-fatigue assessment approach for rotary friction-welded components does not yet exist. The scope of this paper is to present test results for the fatigue behaviour of rotary friction-welded solid shafts made of structural steel S355J2G3 (1.0570) and an approach to fatigue assessment considering residual stress. In contrast to fusion-welded joints, components made by rotary friction welding usually contain compressive residual stress near the weld, which can significantly affect the fatigue strength. For this purpose, specimens were welded and characterised, including metallographic micrographs, hardness measurements, and residual stress measurements. The fatigue tests were performed with a constant amplitude loading in tension/compression or torsion with R = −1. All specimens were investigated without machining of the weld flash, either in the as-welded state or after a post-weld stress-relief heat treatment. In addition, the friction welding process and the residual stress formation were analysed using numerical simulation. The characterisation results are integrated into a fatigue assessment approach. Overall, the specimens perform comparatively well in the fatigue tests and the experimentally observed fatigue behaviour is well described using the proposed local approaches.

## 1. Introduction

Rotary friction welding is a well-established industrial joining process, which allows for reproducible, high-quality welds at comparatively low costs. It is commonly used to weld rotationally symmetric parts in mass production or to weld material combinations that cannot be joined by conventional gas metal arc welding (GMAW) or laser processes. Common friction-welded components are, for instance, airbag inflators, drive shafts, camshafts, ball studs, valves in the automotive industry, jet engine components, and drill pipes and hydraulic plunger rods for the oil field service and for the construction sector. Because no filler materials or special surface preparations are required, rotary friction welding is an ecological welding solution. Direct-drive rotary friction welding can be divided into four process phases according to ISO 15620 [1]: the process starts with a careful contacting of the mating components in the contact phase. In the friction phase, the pre-products are pressed together axially with the friction pressure while one is rotating and the other is stationary. Once sufficient heat and burn-off has been generated, the braking phase is initiated, and the rotation is stopped. This is followed by the forging phase, where an increasing forging pressure is applied and held for a few seconds to ensure the welding [1]. Friction welding differs fundamentally from fusion welding by the absence of a liquid phase in the welding process. It is therefore classified as a solid-state welding process. Since most of the friction-welded components are exposed to cyclic loads during operation, a reliable fatigue life assessment is inevitable for the component design.

To date, diverse studies have assessed the fatigue strength of welded joints with extensive focus on frequently used fusion welding processes such as gas metal arc welding or power beam welding processes. These processes typically exhibit stress concentrations resulting from the weld transition geometry and metallurgical notches [2,3]. It is well understood that these features show a dominant effect on the fatigue strength of fusion-welded joints [2,4]. Thereby, previous studies indicate that the tensile properties of the base material show a subordinate effect on the resulting fatigue strength [5,6,7]. This is attributed to an increasing sensitivity to the geometric notches caused by welding with increasing material strength [5,6,7]. Accordingly, established fatigue assessment methods for welded joints primarily use a classification system based on the present weld detail (FAT class). All fatigue-relevant effects, such as local stress concentrations and material influence, are contained in this assessment using the FAT class according to the nominal stress approach. Different joints therefore require individual FAT classes to account for their distinct characteristics based on experimental data. Local-fatigue assessment approaches, on the other hand, such as the notch stress approach [8] or the theory of critical distances [9,10] are intended to assess the local stress concentration depending on the actual joint geometry, while material effects are considered in a collective FAT class. Therefore, local assessment approaches are local concepts better suited for the evaluation of less-common or complex welding details. However, these methods have been proven to work reliably for fusion-welded joints but have not been verified for friction-welded joints.

A number of studies have examined the fatigue strength of rotary friction-welded steel. Hasegawa et al. investigated the fatigue strength of similar friction-welded carbon steels S15C to S55C [11]. As opposed to fusion-welded joints, a strong influence of the material strength on the fatigue strength was found for friction-welded joints. The fatigue strength reached up to Δσ_N=2×10^6_ ≈ 600 MPa for rotary bending of S55C specimens with flash, with a comparatively shallow slope of the S-N curve between k = 9 and k = 20. The friction-welding parameters were shown to influence the fatigue strength, but the main effect was identified as the shape, and thus the stress concentration factor, of the toe radius [11], which was the topic of further research [12]. For dissimilar joints of 1.4301 and 1.6565, Hascalik measured a fatigue strength of Δσ_N=2×10^6_ ≈ 450 MPa to 600 MPa in a rotary bending test [13]. The influence of residual stress on the fatigue strength of friction-welded joints made from steel similar to 1.1189 was characterised by Manteghi [14]. As-welded joints were found to have higher strength than stress-relieved joints, indicating beneficial compressive residual stress and exceeding the applicable British Standard values for conventional fusion welds. A comparatively high fatigue strength, similar to the base material strength, was also found in [15,16,17]. Priymak also measured compressive residual stress at the surface of friction-welded specimens [18].

Summarizing, previous research reveals that friction welds exhibit substantially higher fatigue strength than fusion welds, indicating that a direct application of common assessment approaches would result in an overly conservative component design. The fundamental differences in process control and influencing variables therefore necessitate further investigation of the key influences on the fatigue properties as well as the conception of a customized fatigue assessment approach for friction-welded joints. Thus, the aim of this research is the following:

- To further investigate the fatigue strength of friction welded joints.
- To determine the influence of residual stress on the fatigue strength.
- To propose a first iteration of a fatigue assessment approach for friction-welded joints with an appropriate safety factor.

Therefore, specimens are manufactured, and characterised regarding microstructure, hardness, and residual stress. Fatigue test results are presented and complemented by a finite element simulation of the welding process and a calculation of the fatigue strength. The proposed assessment approach is further on based the German FKM-guideline: Computational strength proof for machine parts [19] (scope of application: unwelded material), including the influences resulting from residual stress on the fatigue strength. The fatigue life estimated from the numerical analysis is subsequently compared to the fatigue test results for validation.

## 2. Materials and Methods

In this study, the fatigue strength of friction-welded structural steel S355J2+N is analysed. The parts to be joined are machined from bar stock with a nominal diameter of 45 mm. According to a chemical analysis, the chemical composition shown in Table 1 complies with the standard set in DIN EN 10025 [20].

**Figure 1:**
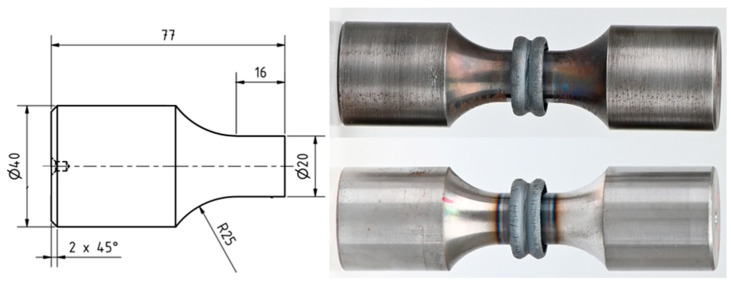
Pre-product geometry and specimens after welding (SR top, AW bottom).

For fatigue testing, the specimens are designed with a testing section and a clamping section, connected by a radius. The clamping section diameter is 40 mm, and the testing section diameter is 20 mm. A 25 mm radius is used for a smooth transition between sections, so that clamping-induced failure in the fatigue tests can be avoided, see Figure 1.

The surface roughness is specified as R_z_ ≤ 6.3 µm. The pre-product geometry aims at having a single circular interface with a symmetric weld flash and heat flux on both sides of the interface while welding. This joint form represents the simplest rotary friction weld and is therefore deemed a suitable starting point for modelling the fatigue strength on a conceptual basis.

In this study, 15 specimens are investigated in the as-welded (AW) condition and 30 specimens after a post-weld stress-relief heat treatment (SR). For the stress-relief heat treatment, specimens are heated and kept at 550–600 °C for a dwell time of 2.5 h. Oxidation protection is provided by flushing the furnace with nitrogen shielding gas. The cooling takes place in the furnace. Additionally, twelve pre-products are stress-relieved prior to welding (Pre-SR), but the resulting specimens are not fatigue-tested. Heat treatment before welding is used to eliminate the residual stress resulting from the machining of the pre-products; thus, the specimens only contain the welding-induced residual stress. These pre-weld stress-relief heat-treated specimens are used for comparing the residual stress measurements to the simulation.

### 2.1. Friction Welding

The friction welding of the specimens was carried out on a computer numerical control friction-welding machine of the type MVR 200 manufactured by H&B Omega Europe GmbH, Sülzetal, Germany. The machine provides a maximum axial force of 200 kN with a vertical axis orientation. The equipment includes a force measurement platform which records the process forces and torques. These are used for force control of the friction welding process and as input data for simulations. The weld specimens are each clamped in a three-jaw chuck.

Welding on this machine is a direct-drive friction welding process. The friction welding parameters correspond to the common values for mild steel, see Table 2 [21]. After a short contact phase, the friction phase takes place, the duration of which is controlled according to the shortening of the welding members. As soon as the weld specimens have reached the pre-set burn-off length of 6 mm, the braking phase is initiated, see Table 2. The forging pressure is applied once the rotational speed during the braking phase falls below 1000 min^−1^. It is then held for 5 s before releasing the specimen.

### 2.2. Friction Welding Simulation

To gain a better understanding of the weld properties and to predict the residual stresses within the welds, the rotary friction welding process was simulated using the proprietary *virtua* RFW R1.023 software developed by Sampro GmbH, Magdeburg, Germany. As the rotary friction welding machine records axial force and the rotational speed throughout the process, this data are used directly as the boundary conditions for the simulation. The simulation uses an axisymmetric approach with linear triangle elements and remeshing [22]. The minimum element size is set to 0.3 mm within the weld interface and increased to 6.0 mm in the clamped region. Regarding the material model, the flow properties are derived from hot tensile tests on a different S355 structural steel carried out in previous studies [23,24,25]. Material strength parameters are available for a higher-strength batch of S355 [23] only. Therefore, the values regarding flow resistance have been scaled down by 25% according to the hardness measurements. The temperature-dependent heat conduction coefficients and heat capacities are provided by [26], the temperature-dependent elastic modulus and thermal expansion coefficients by Eurocode 3 [27].

### 2.3. Hardness and Residual-Stress Measurements

For the evaluation of the resulting microstructure and for hardness measurements, metallographic cross-sections were prepared of the welded joints and analysed on single specimens. Hardness mappings are conducted using an automated ultrasonic contact impedance (UCI) hardness tester with a testing load according to Vickers HV1 and a scan pattern with a point spacing of 0.25 mm. Additionally, conventional hardness measurements according to Vickers HV10 (DIN EN ISO 6507 [28]) are performed along a line with a distance of 1.1 ± 0.1 mm from the surface.

**Figure 2:**
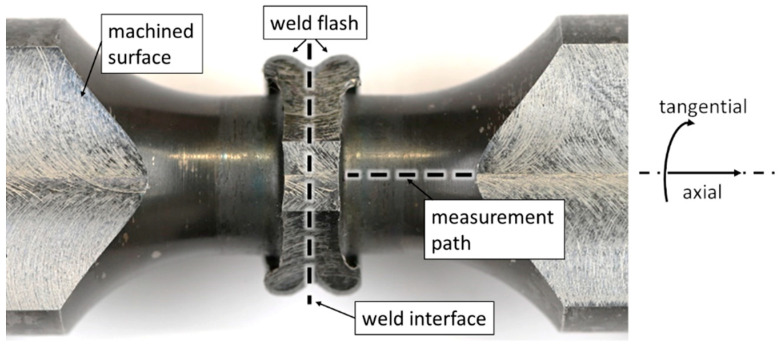
Specimen preparation for residual stress measurement, IA specimen displayed.

Residual stress is measured at the surface in axial and tangential direction along an axial measurement path using the X-ray diffraction method, as displayed in Figure 2.

**Figure 3:**
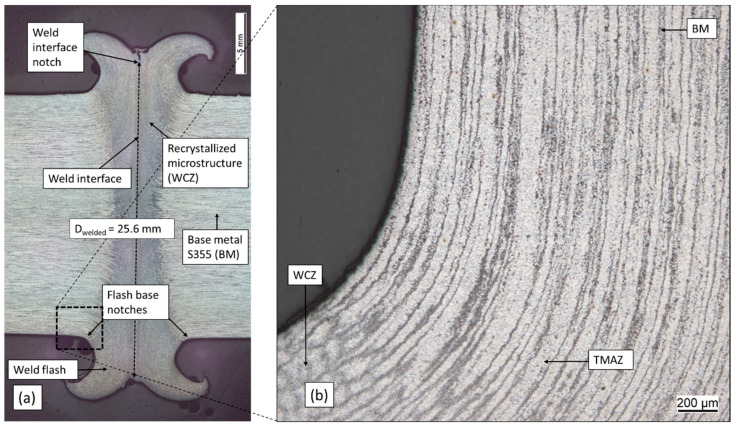
(**a**) Macrograph of welded joint with designations; (**b**) micrograph image of the flash base notch.

To enable measurements close to the weld, a shading of the diffraction signal by the curled weld flash, which can be seen in Figure 3a, must be avoided. Thus, the flash is partially removed by milling. Special care is taken to remove as little material as possible, leaving a stump of the weld flash in place, in an attempt to limit the residual stress redistribution as far as possible.

In total, the residual stresses are measured at several points along the displayed measurement path. The path stretches from a distance of 4.8 mm from the weld interface to 12 mm. For each measurement point, diffraction patterns are obtained at eleven Ψ-angles using copper radiation and a collimator diameter of 2 mm. Residual stress is further determined according to the sin^2^Ψ method.

### 2.4. Fatigue Testing

Fatigue tests were performed under either axial or torsional loading with a constant load amplitude. The stress ratio was set to R = −1, resulting in a mean stress of σ_m_ = τ_m_ = 0 MPa. The stress amplitude was varied between specimens. Servo-hydraulic machines were used to perform testing and to regulate the loading. The test frequency varied in the range 5 Hz ≤ f ≤ 15 Hz, where the lower frequencies were used to minimise heating of the specimen due to high loads. Tests were performed until failure, while recording the cycle numbers, force and displacement, or the torque and twisting angle, respectively. A 5% drop in stiffness as identified from the recorded data is interpreted as the failure point.

## 3. Results

### 3.1. Characterisation of the Welded Specimens

All specimens were welded without irregularities, according to DVS 2909 [21]; the shortening is comparatively constant for all samples with a deviation of ±0.15 mm, and the flash symmetry is consistent, see Figure 1. The welded diameter at the weld interface measures 25.6 mm due to compression during welding, leading to a 64% larger cross-sectional area at the weld interface compared to the nominal cross section, see Figure 3a.

The increased area combined with the distance to the nominal diameter puts the interface notches well out of the load path. The flash base notches appear smooth, and consequently, these welds are rated in class B, according to DVS 2909 [21]. The recrystallised microstructure near the weld interface, with a redirected grain-structure, is clearly visible. The thermo-mechanical working leads to a dynamic recrystallisation, leading to a very fine-grained microstructure, as is typical for friction welds [21]. Previous publications [13,29,30] identify several different microstructural areas in friction welds, with [29] identifying a recrystallised weld centre zone (WCZ), a thermo-mechanically affected zone (TMAZ), a heat-affected zone (HAZ) and the base metal (BM). According to [29], both WCZ and TMAZ are thermo-mechanically affected, resulting in recrystallisation or deformation of the structure, while the HAZ is affected by heat only, and is not deformed. In Figure 3b a distinction of WCZ and TMAZ is possible based on their appearance, but the HAZ is not distinguishable from the base metal in the micrograph. The flash base notches must, by the previous definition, be located at the transition from the thermo-mechanically affected zone to the heat-affected zone.

**Figure 4:**
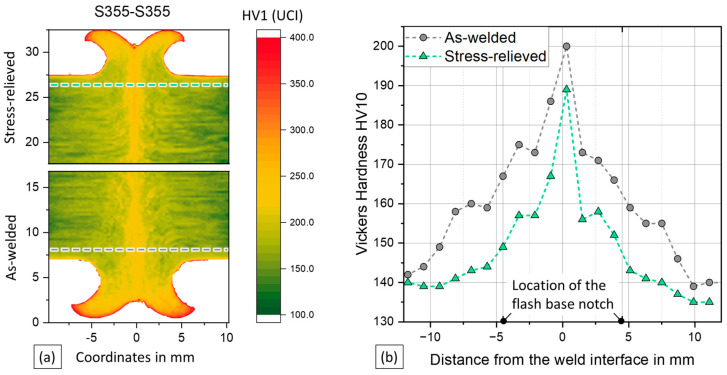
(**a**) Scaled UCI hardness mappings, with half of a stress-relieved specimen displayed on top and half of an as-welded specimen at the bottom; (**b**) Vickers hardness measurements of as-welded and stress-relieved specimens.

The hardness mapping shows an increase in hardness in the weld centre zone and the thermo-mechanically affected zone compared to the base metal, as seen in Figure 4a. The stress-relieved specimen is shown on the top half, while the as-welded specimen is displayed on the bottom side. The UCI hardness measurements are mostly qualitative in nature, because there may be some zero-point drift between different specimens. The shown measurements were scaled to match the reliable HV10 measurements in the base metal area.

As with the micrographs, the heat-affected zone cannot be determined unambiguously from the hardness measurement. The maximum hardness occurs in the weld interface at the outer flash diameter. The measuring line for HV10 measurements displayed in Figure 4b is schematically shown in the figure in part (a). For the as-welded specimens, a base metal hardness of 139 HV10 is determined, while the hardness near the notch (approx. 4.5 mm from the interface) is 167 HV10. Stress relieving reduced the hardness to 135 HV10 and 143 HV10, respectively. A 3% reduction in the base metal was surpassed by a 17% reduction at the flash base notch. Stress-relief heat treatment of steel was observed to lead to hardness loss [31], probably because the stress-relief process reduces the dislocation density and leads to carbon segregation.

### 3.2. Welding and Residual-Stress Simulation

**Figure 5:**
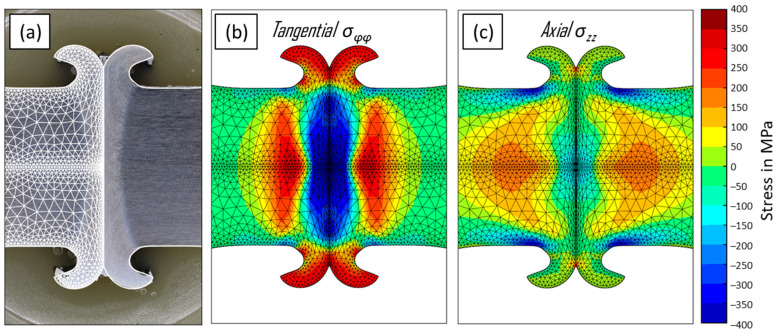
(**a**) Overlay of a macrograph and the outer contour of the FE mesh at the end of the process simulation; (**b**,**c**) residual stresses at simulation end after cooling.

The geometry of the weld zone determined by the simulation model and the actual cross-section obtained from the experiments show good agreement, as depicted in Figure 5a. Closer inspection shows minor differences in the weld interface notch. The interface notches of the model are slightly sharper, which is related to the challenges of modelling bonding and de-bonding in rotary friction welding. The agreement of the weld flash thickness, its overall shape and the weld flash base notches indicate an overall high accuracy of the simulation, validating the approach.

In addition to the weld geometry, the simulation provides results for the residual stress state after welding. The axial residual stresses in the area of the flash base notch are calculated to be compressive at about σ_residual_ = −200 MPa on the surface of the specimen, see Figure 5c. The stress magnitude increases towards the flash base notch. At the centre of the weld interface, compressive axial stresses are calculated. Internally, at approx. 5–10 mm distance from the weld interface, the simulation shows tensile residual stresses. The simulation predicts slight tensile-residual stress at the weld interface notch, though the aforementioned modelling challenges in this area limit the significance of this prediction.

The tangential residual stresses on the surface of the specimen close to the flash base notch are similarly identified as compressive stresses with a comparatively lower magnitude, see Figure 5b. Internal residual stresses are again compressive at the interface and tensile away from it, but at a higher magnitude compared to the axial stress. Significant tensile tangential residual stresses are predicted at the weld interface notch, with the same reservation regarding this result as for the axial stresses. The weld flash is subject to high tensile-residual stress.

### 3.3. Residual Stress Measurements

**Figure 6:**
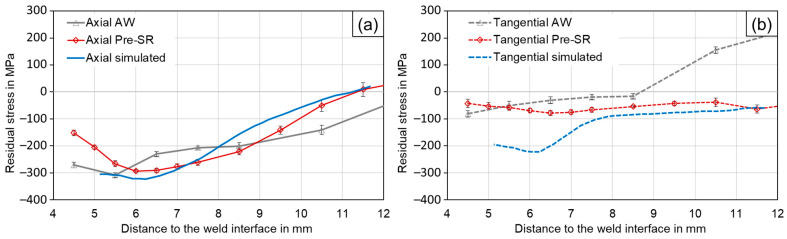
Surface residual-stress distribution measured using X-ray diffraction and extracted from the simulation, shown for (**a**) axial direction, (**b**) tangential direction.

The simulation starts with zero stress in the pre-product, and thus only displays welding-induced residual stresses. Therefore, comparable surface measurements have been conducted using the pre-weld stress-relieved (Pre-SR) specimens for further validation of the simulation. In these, the pre-product machining-related residual stresses were removed by the heat treatment to ensure comparability. Here, the residual stress measurements using the X-ray diffraction method show tangential and axial residual stress in compression, as displayed in Figure 6.

In the range of approx. 7 to 12 mm from the weld interface, the simulation results are in good agreement with the Pre-SR measurements for axial and tangential stress. In this area, the tangential residual stress is around—50 MPa. The axial compressive residual stress starts at roughly −300 MPa at 7 mm and reduces to approximately 0 MPa with increasing distance from the weld interface However, the measured tangential residual stress closer to the weld interface does not exhibit a further increase in compressive stress, contrary to what is predicted by the simulation results. Overall, these results indicate that the methods agree less favourably at the section closest to the flash base notch, where the simulated tangential residual stresses show larger magnitudes. A possible explanation for this is the convex surface of the specimen, leading to deviations of the measured diffraction patterns, particularly for the tangential direction. These measurements are, nonetheless, are to reasonably validate the simulation approach.

Notably, all process-induced residual stresses at the surface of the specimen are measured and predicted by the simulation to be compressive. In [32] it was shown that the cooling of a uniformly heated disk-shaped specimen from the outside can be expected to yield compressive residual stress at the surface with tensile stress in the bulk material, the magnitude of the stress being dependent on the temperature gradient. This may also explain the residual stress distribution in the friction-welded joints, which may possibly be considered as uniformly heated along disk-shaped axial sections, according to their circular symmetrical nature. On every cross-section, the surface cools faster than the interior, mimicking the cooling from the outside, as demonstrated in [32]. This approach would explain the general tendency towards compressive stress at the surface and tensile in the interior, but does not yet explain the compressive residual stress at the weld interface. The simulation results can be used to understand this circumstance: for the tangential residual-stress distribution, a significant impact is likely made by the weld flash. The flash is extruded from the weld area at high temperatures of around 1200 °C [33] and will cool slowest, because of its separated location. Due to the cooling, significant shrinkage is to be expected, which will be resisted by the colder material below the flash. This constraint results in high tensile stress in the weld flash and in compressive stress in the area below the flash. The magnitude of this effect is supposed to be larger than the first-explained mechanism, superimposing the results shown by the simulation in Figure 3.

For further information, the as-welded (AW) specimens are added to the comparison in Figure 6. These represent the specimens which are subject to fatigue testing. Their measurements show a slightly different trend: the tangential results at a distance of more than 9 mm from the weld interface especially, tend towards significant tensile residual stress. This residual stress likely results from the machining of the pre-products, and was not impacted by the welding process due to the distance from the weld.

### 3.4. Fatigue Strength Evaluation

#### 3.4.1. Axial Testing Results

**Figure 7:**
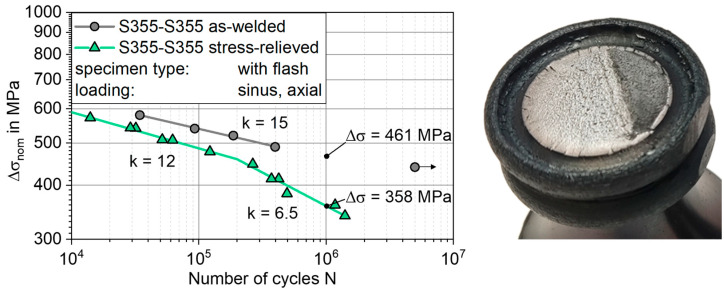
S-N diagram derived for axial fatigue testing and fracture surface of a failed specimen with notch-induced failure.

Under axial fatigue loading, all specimens showed a coherent failure mode, with crack initiation from the notch between the base material and flash, see Figure 7.

This failure characteristic can be attributed to the stress concentration in the flash base notch. Comparing the S-N curves in the as-welded and stress-relieved condition, the as-welded specimens show higher fatigue strength than the stress-relieved specimens, see Figure 7. An endurable stress range of the as-welded specimens at N = 10^6^ cycles can be extrapolated from the data points as Δσ_AW_ = 461 MPa. The slope of the S-N curve is k_AW_ = 15. In contrast, the endurable stress for the stress-relieved specimens is identified as Δσ_SR_ = 358 MPa. The S-N curve of the stress-relieved specimens is bilinear, with a knee point at N = 2 × 10^5^ cycles. The slope for the N ≤ 2 × 10^5^ cycles is k_1SR_ = 12 and the one for N ≥ 2 × 10^5^ cycles is k_2SR_ = 6.5. Thus, the stress relieving resulted in a 29% reduction in fatigue strength. The scatter of the data is 1:T_s_ = 1.06, which is notably low.

#### 3.4.2. Torsional Testing Results

**Figure 8:**
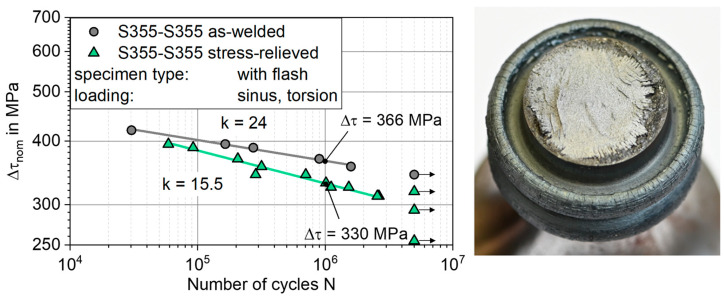
S-N diagram derived for torsional fatigue testing next to a failed specimen with nominal section failure.

Under torsional loading, two distinct S-N curves were determined, as well. The as-welded specimens showed higher fatigue strength, see Figure 8.

Their fatigue strength is Δτ_AW_ = 366 MPa at N = 10^6^, while the test of the stress-relieved specimens resulted in Δτ_SR_ = 330 MPa, which amounts to a difference of approx. 11%. Additionally, the slope of the S-N curve is steeper for the stress-relieved specimens with k_SR_ = 15.5 compared to the as-welded specimens with k_AW_ = 24. The scatter of the data is 1:T_s_ = 1.04.

By analysing the fractured surface, it was found that fatigue failure started either at the flash base notch or at the heat-affected zone/base metal. As no clear distinction could be made between heat-affected zone and base metal on the basis of the cross-sections and hardness measurements (Section 3.1), no further differentiation was made between the crack origins, in this respect. In detail, ten of the fourteen stress-relieved specimens failed from the flash base notch, one in the nominal diameter section, and three specimens were runouts. For the as-welded specimens, three of the six specimens failed in the nominal section, two close to the flash base notch, and one was a runout. The stress relieving thus increased the likelihood of failure at the flash base notch.

#### 3.4.3. Summary of Testing

The testing showed a significant influence of the specimen’s heat-treatment state on the fatigue strength. Comparing the fatigue strength of the friction welds in the as-welded state Δσ_AW,N=2·10_^6^ = 440 MPa with a fusion-welded butt joint, for example Δσ_A-1,N=2·10_^6^ = 190 MPa in [34] or the applicable FAT 90, the high fatigue strength of the friction-welded joint becomes obvious. Additionally, the S-N curve slope is very flat, resulting in this high fatigue strength in the high-cycle regime. Two distinct failure modes were observed:

1. Failure due to the weld flash base notches;
2. Nominal section failure in the base metal or heat-affected zone.

The intended analytical fatigue estimation approach, therefore, needs to include assessment variations for both a notched case and a location without any geometrical notches. Additionally, an influence of the local hardness and residual stress should be included. For a comprehensive consideration of the experimentally determined boundary conditions, the following approach is based on the FKM guideline: computational strength proof for machine components [19]. This allows for simultaneous consideration of material strength, geometry, and mean stress, based on a local approach.

### 3.5. Assessment Approach

As shown in the experiments, the friction-welded joints exceeded the fatigue strength that can be described by nominal stress concepts based on specific notch cases (Max. FAT 160 [35]) by a considerable margin. According to the characterisation, the friction welds did not display the seam properties modelled with the notch cases (HAZ, sharp notches, tensile residual stress). An assessment of GMAW-welded joints with the FKM guideline would also use notch cases. Since the modelling of the friction-welded joints requires material, geometry and mean stress influences to be incorporated, the joint had to be treated as base material. The calculation of fatigue strength according to the presented approach proceeds as follows:

3. Estimate the base fatigue strength of the material.
4. Derive a local endurable stress amplitude (at R = −1, N = 10^6^, p_surv_ = 97.5%) via a support effect calculation including local stress gradient, notch sharpness, roughness, base material strength, and surface factors.
5. Scale the local endurable-stress amplitude to a different R-ratio for residual stress-influence consideration.
6. Calculate endurable nominal-stress range from the endurable local-stress amplitude.
7. Construct the design curve from the endurable stress range and the slope.

#### 3.5.1. Calculation of the Local Material Strength

According to the FKM guideline, the endurable stress amplitudes σ_w_ for structural steels at N = 10^6^ under normal stresses can be derived from the ultimate tensile stress R_m_ with Equation (1).

σ_w_ = 0.45 × R_m_ (1)

τ_w_ = 0.577 × σ_w_ (2)

The endurable stress amplitude under torsional loading τ_w_ can be estimated by Equation (2), based on the von Mises yield criterion. In order to perform a differentiated assessment regarding local distinctions, the information on the tensile strength has to be local to the assessment location. In this respect, the local tensile strength was estimated from the Vickers hardness in accordance with DIN EN ISO 18265 [36], using Equation (3).

R_m_ ≈ (3.2 × HV10) × MPa (3)

The influence of the stress gradients at the notched areas is considered by the Siebel–Stieler approach from the FKM guideline [37]. For this purpose, the stress gradient at the surface G_σ_ is evaluated according to Equation (4) and a support factor n is calculated with Equation (5). The input data for Equation (4) was derived using the Abaqus finite element software with the geometry of the macrograph and a linear elastic-material model for steel (E = 210 GPa, ν = 0.3). One finite element model was used to describe all specimens, since no significant variations in geometry were observed between the specimens.

G_σ_ = 1/Δs × δσ/σ_max_ (4)

n = f(G_σ_, R_m_) (5)

This support factor n plus additional factors for the notch effect K_f_ and the influence of surface roughness K_R_ are used to calculate a construction factor K_WK_, as given by Equation (6). Other factors used by the FKM guideline are not applicable here and are therefore omitted. The local endurable-stress amplitude σ_WK_ at N = 10^6^ is then calculated from the endurable stress amplitude σ_w_ and the construction factor K_WK_, see Equation (7). The same approach applies to torsional loading.

K_WK_ = 1/n × [1 + ((1/K_f_) × (1/K_R_ − 1))] (6)

σ_WK_ = σ_w_/K_WK_ (7)

#### 3.5.2. Influence of Residual Stress

The FKM guideline does not provide the means to calculate the influence of residual stress directly, but since residual stresses may be considered as mean stresses if the static- and cyclic-yield stress is not exceeded [38], the given mean stress approach can be applied. For the specifics of a mean stress application, the cyclically stable stress level must be determined independently of the guideline specifications.

The local endurable amplitude for a stress ratio σ_AK_ different from R = −1 can be calculated to the approximate residual stresses, according to Equation (8), with the factor K_AK_ being calculated using the equation specified as most relevant to the practice in the guideline [19]. Due to the high levels of residual stresses in the specimens, a simple equation is used to approximate the stable residual stress, depending on the yield strength R_p0.2_. The equation is based on the assumption that the residual stresses are only stable if no plastic deformation occurs due to the addition of residual stress and load amplitude. If plastic deformation occurs, the new stable residual-stress level is assumed to be reduced by the first plastification in such a way that no yielding will occur on subsequent cycles. This introduces an upper limit for the stable residual stress, according to Equation (9). For this calculation, the yield ratio is assumed to be 0.7, see Equation (10).

σ_AK_ = K_AK,σ_(σ_residual,stable_) × σ_WK_ (8)

σ_residual,stable_ = min(R_p0.2_ − σ_WK_ or σ_residual_) (9)

R_p0.2_ = R_m_ × 0.7 (10)

Finally, the local endurable amplitude at the chosen stress ratio σ_AK_ has to be transformed to a global stress value (nominal stress space) to enable a comparison between different locations and specimens. This is performed by dividing σ_AK_ by the local stress-concentration factor K_t_ and multiplying by 2 to obtain the stress range, see Equation (11).

Δσ_concept_ = 2 × σ_AK_/K_t_ (11)

#### 3.5.3. Constructing the S-N Curve

When comparing the fatigue strength, both the calculated and the experimental testing data should have the same survival probability. Since the scatter of the fatigue test data is notably low, even lower, in fact, than the typical scatter included in the calculation concept’s base data, the comparison would be overly conservative. Hence, the fatigue test data are not converted to a survival probability of 97.5%, but the FKM-calculated data are converted to p_surv_ = 50%, assuming a concept scatter of 1:T_s_ = 1.25. This results in a scaling factor of 1.2 to obtain Δσ_concept, 50%_.

From the Δσ_concept, 50%_ stress range at N = 10^6^, an S-N curve is constructed via a specified slope. The FKM guideline states a slope of k_σ_ = 5 for axial load and k_τ_ = 8 for shear load. Due to the low slopes of the friction-welded joints, as shown in Section 3.4, these specifications have to be modified, particularly for friction-welded joints. In this case, the used slopes are k_σ,RFW_ = 10 for axial load and k_τ,RFW_ = 15 for shear load.

#### 3.5.4. Estimation Results

According to the FKM guideline, compressive residual stresses will lead to an increase in fatigue strength under axial loading. On the other hand, any mean stress will introduce a reduction of the torsional fatigue strength, since the absolute value of the residual stress is used for the calculation of the factor K_AK,τ_ [19].

The calculation results for critical locations (flash base notch, nominal section) and major input data are listed in Table 3.

The results are first distinguished by the two failure locations notch and nominal section and then further distinguished by either torsional- or axial-loading direction. From top to bottom, there are two major sections according to the specimen condition: SR and AW. Within these divisions, the hardness, the calculated fatigue strength, and the tested fatigue strength are listed (both at N = 10^6^ and p_surv=50%_). An additional hypothetical result is listed for the AW condition, without consideration of the residual stress. This result allows for a differentiation of the respective hardness and residual-stress influence.

Looking at the column for notch, axial, the calculated fatigue strength for SR specimens (325.5 MPa) is 9% lower than the test result (358 MPa). For the AW specimens, the calculated strength is 20% lower than that determined by the experiments. Nonetheless, the fatigue strength is predicted to be reduced by 12%, due to the stress-relief heat treatment. This 12% is split into 3% loss due to lost compressive residual stress and 9% loss due to hardness. Therefore, the compressive residual stresses yield an increase in fatigue strength. With all calculated strengths lower than the test results, the estimation is conservative.

Considering the data for notch, torsion, the calculated fatigue strength for SR specimens is 21% lower than the test result. For the AW specimens the calculated strength is also 21% lower than that determined in the experiments. Here, the predicted loss is 9%, due to the stress-relief heat treatment from AW to SR, which results from a 2% increase due to relieved compressive-residual stress counteracted by 11% of strength loss due to reduced hardness. The estimation is conservative in this case, too.

For the nominal section under axial loading, no experimental test data could be obtained, due to occurrence of failure only at the flash base notch. However, the calculated fatigue strength is considerably higher at the nominal section compared to the notch location, confirming that failure is to be expected in the notch area.

Lastly, looking at nominal section, torsion, the fatigue strength is underestimated by 15% for the SR specimens and by 22% for the AW cases. The calculated strength loss due to stress-relief heat treatment is low, at 1%, split into a 2% increase due to stress-relief and counteracted by a 3% loss due to reduced hardness.

Comparing between columns, the differences between notch, torsion and nominal section, torsion are interesting: in the AW case, the predicted strengths for notch and nominal section are nearly identical, with 287.6 MPa and 285.2 MPa. Conversely, in the SR case, the notch is clearly the location with lower fatigue strength (260.3 MPa versus 281.9 MPa). This highlights a predicted switch from an ambiguous failure location to clearly notch-related failure following the stress-relief heat treatment. In the axial load case, the notch is distinctly predicted as failure-critical for AW and SR states.

## 4. Discussion

**Figure 9:**
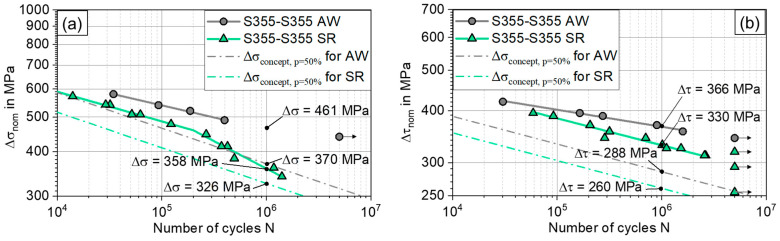
Fatigue testing results compared to the calculated prediction; 50% survival probability shown for (**a**) axial loading, (**b**) torsional loading.

As summarized in Figure 9, the calculated fatigue strengths are found to be generally lower than the experimentally observed ones.

**Figure 10:**
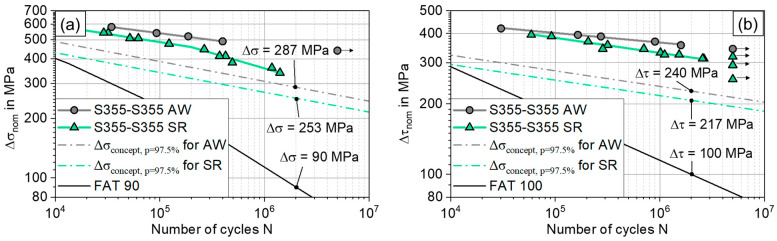
Fatigue testing results compared to the proposed assessment and assessment via the FAT class for a butt-welded joint [35]; 97.5% survival probability shown for (**a**) axial loading, (**b**) torsional loading.

This leads to a conservative estimation of the fatigue strength, even though all the compared values are for p_surv_ = 50%. The best match between predicted and determined fatigue strength was found for the stress-relieved specimens under axial loading, due to the bilinear S-N curve. Especially for torsional loading, the fatigue strength is still underestimated by 21% using the proposed approach, which would lead to an overly conservative component design. However, comparing the presented approach to a nominal stress concept approach leads to a favourable outlook: for the axially loaded AW specimens, FAT 90 could be considered an appropriate design value [35], yielding Δσ_N=2×10^6, 97.5%_ = 90 MPa as acceptable, compared to Δσ_N=2×10^6, 97.5%_ = 287 MPa with the presented approach, visible in Figure 10a. This represents a tripling of the acceptable fatigue loading for the friction-welded joint. In the torsional case, FAT 100 could be applied according to [35], whereas the presented approach proposes Δτ_N=2×10^6, 97.5%_ = 217 MPa, as shown in Figure 10b.

The significantly lower fatigue-strength estimation using design concepts for fusion welds [35] might result from several differences to friction-welded joints: a weakened heat-affected zone, e.g., recognisable by the coarse-grain zone as known from GMAW, which is not commonly observed for friction welds. Fusion welding also usually introduces significant tensile stress near the weld, whereas the friction welds were shown to result in compressive residual stress. Arc welds are allowed to have some undercut and excess weld metal, while the flash base notch of the presented welds is smoothly transitioning from the nominal section into the weld flash, reducing the notch effect. With a mild notch at the flash base, the process-induced hardness increases at this location, and even leads to further strength improvement.

The reduction in the fatigue strength from stress relieving observed in the experiments under torsion is well matched by the calculated results, but the calculation is overly conservative, in general. Nevertheless, the prediction of the failure-critical location matches the experiments, showing either the notch or the base metal as critical for torsion. The experimentally observed shift towards failure near the notch following the stress relieving is correctly predicted in the calculated data. Possible explanations for the remaining conservativity of the proposed approach are the following:

- The surface roughness was specified for specimen preparation and therefore assumed to be R_Z_ = 6.3 µm in the calculation. No further measurements on the roughness were conducted, due to poor accessibility at the flash base notch. Therefore, lower actual roughness could have improved the tested fatigue strength.
- The estimation of the local tensile strength based on hardness measurements might involve inaccuracies, affecting further calculations.
- Regarding the influence of residual stress on the fatigue strength, the calculation is likely limited by not considering residual stress relaxation. In reality, the mechanisms for residual stress relaxation are very complex, especially under cyclic loading. Nevertheless, the FKM guideline already approximates the trends in axial strength well.

Regarding the reliability of the fatigue testing data, the number of AW specimens was considerably small. Fortunately, the scatter of the data was very low across all friction-welded joints, which is why the testing results are still considered as accurate.

The process and residual-stress simulation was shown to be capable of re-creating the weld flash accurately. Additionally, the simulated residual-stress results match the trend of compressive stresses in an axial and tangential direction near the weld. From a 7 mm distance to the weld interface upwards, the magnitude of the residual stress was accurate. The offset closer to the flash base notch may be related to removal of the weld flash for accessibility and the difficulties of measuring residual stress near the non-planar notch using X-ray diffraction. Also, node-based data are compared to experimental data averaged over a 2 mm diameter circle. For thermo-mechanical simulations, the material-model base data are hard to obtain, which is why a previously obtained model was modified to match the hardness of this research. Hence, the material model was not validated specifically for the specimen material in this study.

## 5. Conclusions

The tested friction-welded joints were found to exhibit very high fatigue strength in the high-cycle fatigue regime, considerably exceeding commonly applicable design values used for fatigue evaluation of fusion welds based on nominal stress concepts at N = 2 × 10^6^. In addition, the experiments showed S-N curves with a remarkably flat slope, k = 6.5 to k = 15 for axial load and k = 15.5 to k = 24 for torsional load, and the stress-relief heat treatment steepened the slope. Reasons for the high fatigue strength are determined to be a mild-fatigue critical notch at the weld flash base, combined with a moderately increased local hardness and corresponding strength at this location. This study set out to determine the influence of residual stress on the fatigue properties based on experimental fatigue testing in combination with a local-fatigue assessment approach, specifically adapted to the requirements of friction-welded joints. The following key findings were derived from this method:

- The experimental and numerical determination of residual stress has shown that compressive residual stress is found in the failure-critical area of the flash base notch. Tensile residual stress is present on the inside of the specimen. Adding to surface-sensitive measurements, the numerical analysis can provide information on the global residual-stress distribution.
- Fatigue testing under torsional and axial load confirmed the high fatigue strength of friction-welded joints, known from the literature. In this regard, the S-N curves generally exhibited a flat slope and low scatter.
- An approach based on the FKM guideline is suitable for providing a conservative fatigue assessment for friction-welded joints, taking into account local geometry, strength, and residual stress. Key changes have to be made to the slope of the S-N curve: k_σ,RFW_ = 10 for axial load and k_τ,RFW_ = 15 for shear load.
- The influence of residual stress on the fatigue of friction-welded joints was determined to depend on the load case. In this regard, the presence of residual stress was found to have a positive impact under axial loading, while reducing the strength in torsion.
- In the case of the presented joints, the hardness at the failure-critical location, the flash base notch, is increased by the process. The locally increased hardness increases the overall fatigue strength of the part, even outweighing the detrimental effects of residual stress in torsion.

Overall, this study contributes to the understanding of the fatigue of friction-welded joints. For the first time, a local-fatigue assessment approach is established. Future practical applications could include a theoretical weld design using numerical process simulation to provide local geometry and material properties and stress distribution, in combination with the proposed approach to estimate the fatigue strength. On this basis, only a low number of welds would be manufactured and tested to confirm the design. This process would consequently reduce the need for expensive experimental fatigue testing, opening up potential for improved lightweight and efficient design, with friction-welded joints and their comparatively high strength, shown in Figure 10. However, further studies need to be carried out in order to validate the approach for other geometries, materials and loads.

## Figures and Tables

**Table 1:** Chemical composition of the tested S355J2+N.

Element	C	Si	Mn	P	S	Cr	Mo
wt. %	0.169	0.173	1.206	0.011	0.026	0.106	0.019
	Ni	Al	Nb	Ti	V	Cu	N
	0.044	0.015	0.0038	0.015	0.0037	0.133	0.013

**Table 2:** Friction welding parameters.

Contact Phase	Friction Pressure	Burn-Off Length	Rotational Speed	Forging Pressure	Forging Time
0.5 s	80 MPa	6 mm	1900 min^−1^	160 MPa	5 s

**Table 3:** Fatigue strength estimation results, strength specifications with 50% survival probability.

		Notch		Nominal Section	
		Torsion	Axial	Torsion	Axial
SR	Stress concentration factor K_t_	1.30	1.78	1	1
	Hardness HV10	143	149	135	
	Δσ_concept, 50%_ or Δτ_concept, 50%_ in MPa	260.3	325.5	281.9	441.3
	Fatigue test result in MPa	330	358	330	-
AW	Stress concentration factor K_t_	1.35	1.89	1	1
	Hardness HV10	167		139	
	σ_residual,stable_ in MPa	−51.7	−91.5	−58.9	−122.5
	Hypothetical: Δσ_concept, 50%_ or Δτ_concept, 50%_ with no residual stress in MPa	292.2	359.3	289.7	453.3
	Δσ_concept, 50%_ or Δτ_concept, 50%_ in MPa	287.6	369.7	285.2	470.3
	Fatigue test result in MPa	366	461	366	-
	Conservative estimation	yes	yes	yes	no data

## Data Availability

The raw data supporting the conclusions of this article will be made available by the authors on request.

## Abbreviations

**Symbol**	**Unit**	**Description**
AW		Specimens in the as-welded state
FAT		Classification reference to the endurable stress range, according to [35]
GMAW		Gas metal arc welding
SR		Specimens that have been stress-relieved after welding
Pre-SR		Specimens that have been stress-relieved prior to welding
BM		Base metal
HAZ		Heat-affected zone
TMAZ		Thermo-mechanically affected zone
WCZ		Weld centre zone
D_welded_	mm	Welded diameter at the weld interface
f	1/s	Frequency of the fatigue test
G_σ_	MPa/mm	Referenced stress gradient according to the FKM guideline
K_AK_ (K_AK,σ_ or K_AK,τ_)	- -	Mean stress factor according to the FKM guideline, normal or shear stress
K_WK_ (K_WK,σ_ or K_WK,τ_)	- -	Construction factor according to the FKM guideline, normal or shear stress
K_f_	-	Estimated notch factor according to the FKM guideline
K_R_	-	Roughness factor according to the FKM guideline
K_t_	-	Stress concentration factor according to the FKM guideline
k	-	Slope of the S-N Curve
k_σ_ k_τ_	- -	Slope of the S-N curve according to the FKM guideline, normal or shear stress
k_σ,RFW_ k_τ,RFW_	- -	Proposed slope of the S-N curve for friction-welded joints, normal or shear stress
N	-	Number of cycles in the fatigue test
n	-	Support effect number according to the FKM guideline
p_surv_	-	Survival probability
R	-	Stress ratio
R_m_	MPa	Ultimate tensile strength
R_p0.2_	MPa	Yield strength
R_Z_	µm	Surface roughness parameter
Δs	mm	Distance from the neighbouring point to the surface according to the FKM guideline
δσ	MPa	Stress delta between the surface and the neighbouring point according to the FKM guideline
σ_max_	MPa	Maximum stress at the surface according to the FKM guideline
σ_residual_	MPa	Residual stress
σ_w_ τ_w_	MPa MPa	Endurable stress amplitude of the material at N = 10^6^ according to the FKM guideline, normal or shear stress
σ_WK_ τ_WK_	MPa MPa	Local endurable-stress amplitude at N = 10^6^ according to the FKM guideline, normal or shear stress
σ_AK_ τ_AK_	MPa MPa	Local endurable-stress amplitude for differing stress ratio at N = 10^6^ according to the FKM guideline, normal or shear stress
σ_residual,stable_	MPa	Estimated stable residual-stress level after the first cycle
σ_m_ τ_m_	MPa MPa	Mean stress, normal or shear stress
Δσ Δτ	MPa MPa	Stress range, normal or shear stress
Δσ_AW_, Δσ_SR_ Δτ_AW_, Δτ_SR_	MPa MPa	Experimentally measured fatigue strength for differing specimen and load conditions
Δσ_concept_	MPa	Endurable nominal-stress range at N = 10^6^
Δσ_concept, 50%_ Δτ_concept, 50%_	MPa MPa	Calculated fatigue strength at N = 10^6^, 50% survival probability, normal or shear stress
Δσ_N = 2×10^6, 97.5%_ Δτ_N = 2×10^6, 97.5%_	MPa MPa	Calculated fatigue strength at N = 2 × 10^6^, 97.5% survival probability, normal or shear stress
1:T_s_	-	Scatter of the S-N curve

## References

[1] (2019). Welding—Friction Welding of Metallic Materials (German Version).

[2] Maddox S.J. (1974). Assessing the significance of flaws in welds subject to fatigue. Weld. J..

[3] Braun M., Baumgartner J., Hofmann G., Drebenstedt K., Bauer N., Bakhschi H., Kuhlmann U. (2023). A statistical assessment of the fatigue strength improvement of butt-welded joints by flush grinding. Weld. World.

[4] Liinalampi S., Remes H., Romanoff J. (2019). Influence of three-dimensional weld undercut geometry on fatigue-effective stress. Weld. World.

[5] Sonsino C.M. (2007). Light-weight design chances using high-strength steels. Mater. Werkst..

[6] Braun M., Ahola A., Milaković A., Ehlers S. (2022). Comparison of local fatigue assessment methods for high-quality butt-welded joints made of high-strength steel. Forces Mech..

[7] Sonsino C.M., Bruder T., Baumgartner J. (2010). S-N lines for welded thin joints—Suggested slopes and FAT values for applying the notch stress concept wot various reference radii. Weld World.

[8] Baumgartner J. (2017). Review and considerations on the fatigue assessment of welded joints using reference radii. Int. J. Fatigue.

[9] Taylor D. (1999). Geometrical effects in fatigue: A unifying theoretical model. Int. J. Fatigue.

[10] Baumgartner J., Schmidt H., Ince E., Melz T., Dilger K. (2015). Fatigue assessment of welded joints using stress averaging and critical distance approaches. Weld. World.

[11] Hasegawa M., Ieda T., Taki N. (1997). Fatigue strength of friction welded joints with flash in various carbon steels. Weld. Int..

[12] Hasegawa M., Ieda T., Asada T., Taki N. (1997). Effects of the toe shape of the flash on stress concentration factor in friction welded joints. Weld. Int..

[13] Hascalik A., Ünal E., Özdemir N. (2006). Fatigue behavior of AISI 304 steel to AISI 4340 steel welded by friction welding. J. Mater. Sci.

[14] Manteghi S., Gibson D., Johnston C. Fatigue of Friction Welds Manufactured in Air or Underwater. Proceedings of the ASME 2017 36th International Conference on Ocean, Offshore and Arctic Engineering.

[15] Neumann A. (1982). Berechnung von Reibschweißverbindungen. Schweißtechnik.

[16] Sahin M. (2005). Joining with friction welding of high-speed steel and medium-carbon steel. J. Mater. Process. Technol..

[17] Murti K.G.K., Sundaresan S. (1986). Structure and properties of friction welds between high-speed steel and medium-carbon steel for bimetal tools. Mater. Sci. Technol..

[18] Priymak E., Boumerzoug Z., Stepanchukova A., Ji V. (2020). Residual Stresses and Microstructural Features of Rotary-Friction-Welded from Dissimilar Medium Carbon Steels. Phys. Met. Metallogr..

[19] Forschungskuratorium Maschinenbau FKM (2012). Rechnerischer Festigkeitsnachweis für Maschinenbauteile.

[20] (2019). Hot Rolled Products of Structural Steels—Part 2: Technical Delivery Conditions for Non-Alloyed Structural Steels. German Version.

[21] DVS—Deutscher Verband für Schweißen und Verwandte Verfahren e. V (2009). DVS 2909-1, -2, -3, -4, -5. Reibschweißen von Metallischen Werkstoffen.

[22] Schmicker D. (2015). A Holistic Approach on the Simulation of Rotary Friction Welding. Ph.D. Thesis.

[23] Schmicker D., Paczulla S., Nitzschke S., Groschopp S., Naumenko K., Jüttner S., Strackeljan J. (2015). Experimental identification of flow properties of a S355 structural steel for hot deformation processes. J. Strain Anal. Eng. Des..

[24] Rößler C., Schmicker D., Naumenko K., Woschke E. (2018). Adaption of a Carreau fluid law formulation for residual stress determination in rotary friction welds. J. Mater. Process. Technol..

[25] Rößler C., Schmicker D., Sherepenko O., Halle T., Körner M., Jüttner S., Woschke E. (2020). Identification of the Flow Properties of a 0.54% Carbon Steel during Continuous Cooling. Metals.

[26] Wichers M. (2006). Schweißen Unter Einachsiger, Zyklischer Beanspruchung Experimentelle und Numerische Untersuchungen. Ph.D. Thesis.

[27] (2022). Eurocode 3: Design of Steel Structures—Part 1–2: General Rules.

[28] (2018). Metallic Materials—Vickers Hardness Test–Part1: Test Method (German Version).

[29] McAndrew A.R., Flipo B.C.D. Linear Friction Welding for Near Net Shape Manufacturing of Titanium Alloy Ti-6Al-4V Aerospace Components. Proceedings of the 2018 9th International Conference on Mechanical and Aerospace Engineering (ICMAE).

[30] Rehman A.U.C., Babu N.K., Talari M.K., Usmani Y.S., Khalefah H.A. (2021). Microstructure and Mechanical Property Correlation Between Rotary Friction Welded Nitinol–Nitinol Joints. Front. Mater..

[31] Evans G. The Effect of Stress Relieving on the Microstructure and Properties of C-Mn All-Weld Metal Deposits. Proceedings of the 65th Annual AWS Meeting.

[32] Liu X., Luzin V., Qin H., Bi Z., Li m., Liu Y., Sun K., Chen D. (2022). Mapping of three-dimensional residual stresses by neutron diffraction in nickel-based superalloy discs prepared under different quenching conditions. Mater. Today Commun..

[33] Akram J., Kalvala P.R., Jindal V., Misra M. (2018). Evaluating location specific strain rates, temperatures, and accumulated strains in friction welds through microstructure modeling. Def. Technol..

[34] Kranz B., Sonsino C.M. (2010). Verification of FAT values for the application of the notch stress concept with the reference radii r(ref) = 1.99 and 0.05 mm. Weld World.

[35] Hobbacher A. (2019). Recommendations for Fatigue Design of Welded Joints and Components.

[36] (2014). Metallic Materials—Conversion of Hardness Values (German Version).

[37] Siebel E., Stieler M. (1955). Ungleichförmige Spannungsverteilung bei Schwingender Beanspruchung. VDIZ.

[38] Kobelev V. (2023). Effects of Mean Stress and Multiaxial Loading on the Fatigue Life of Springs. Eng.

